# The challenges of voluntary care provision for hospitalized patients with COVID‐19: A qualitative study of the public volunteers' experiences

**DOI:** 10.1111/hex.13998

**Published:** 2024-02-25

**Authors:** Fatemeh Karami, Alireza Nikbakht Nasrabadi, Camellia Torabizadeh, Monir Mazaheri, Leila Sayadi

**Affiliations:** ^1^ Department of Medical and Surgical Nursing, School of Nursing and Midwifery Tehran University of Medical Sciences Tehran Iran; ^2^ Department of Medical and Surgical Nursing, Community Based Psychiatric Care Research Center, School of Nursing and Midwifery Shiraz University of Medical Sciences Shiraz Iran; ^3^ Department of Nursing Sciences Sophiahemmet University Stockholm Sweden; ^4^ Department of Neurobiology, Care Sciences and Society Karolinska Institutet Stockholm Sweden; ^5^ Nursing and Midwifery Care Research Centre, School of Nursing and Midwifery Tehran University of Medical Sciences Tehran Iran

**Keywords:** challenges, content analysis, COVID‐19, public volunteers, qualitative research

## Abstract

**Introduction:**

During the COVID‐19 pandemic, there was a remarkable increase in public volunteering for the care of hospitalized patients. They faced challenges during their voluntary care provision. This study aimed at exploring public volunteers' experiences of the challenges of the voluntary care provision to hospitalized patients with COVID‐19.

**Methods:**

A descriptive qualitative study with an inductive content analysis method was conducted, 2022–2023. Eighteen public volunteers providing care to hospitalized patients with COVID‐19 were purposefully selected among 10 hospitals, specialized in COVID‐19 care in Tehran and Shiraz, Iran. Data were collected over 7 months through in‐depth semistructured interviews and concurrently analyzed using conventional content analysis methods.

**Findings:**

The challenges of voluntary care provision to hospitalized patients with COVID‐19 were illustrated in five main categories, each with two subcategories. The categories included structural challenges, interpersonal conflicts, financial constraints, covert participation and the deteriorating condition of care provision. The subcategories comprised lack of volunteer recruitment bases, ineffective organization of voluntary activities, pervasive distrust, heightened risk of clinical errors, conflicts between volunteer commitments and primary occupation, lack of financial support, lack of family support, isolation by friends, mental trauma and physical exhaustion.

**Conclusion:**

Public volunteers encounter diverse challenges while providing care to hospitalized patients with COVID‐19, which negatively impacts their motivation to serve. By addressing these challenges, we can create a more supportive environment for volunteers and enhance the quality of care provided to patients during public health emergencies. Identifying such challenges can assist healthcare managers and policymakers develop effective strategies to mitigate mounting difficulties and enhance volunteer services, thereby improving the overall quality of care provided to patients during public health crises.

**Patient Contributions:**

Participants were identified and recruited after the study objectives were explained in person to the managers. The participants were approached and interviewed by one author. Participation was voluntary and the participants did not receive any financial compensation for their time.

## 1 INTRODUCTION

In December 2019, a new type of human coronavirus emerged, leading to the onset of coronavirus disease 2019 (COVID‐19).^1^ Its rapid spread prompted the World Health Organization to declare COVID‐19 as a pandemic.^2^ As of 4 November 2023, the global tally of COVID‐19 cases surpassed 771 million, with nearly 7 million reported deaths attributed to the disease.^3^

The COVID‐19 pandemic significantly increased the workload of healthcare providers and triggered severe staff shortages due to the substantial rise in the number of affected patients.^4^ For instance, in Iran, the number of nurses per hospital bed decreased from 0.9 to 0.7 after the onset of the pandemic.^5^

Utilizing volunteers within healthcare systems is a key strategy to enhance community health during crises such as pandemics.^6^ Reviewing the literature indicates that engagement in voluntary services to combat COVID‐19 has produced positive outcomes in numerous countries. For example, in China, volunteers contributing their money and other resources, including medical supplies to support the health system' efforts in fighting against the virus and saving as many lives as possible.^7^ In the United Kingdom, volunteer groups played a crucial role in organizing and managing the aftermath of the disease within the community. They facilitated the delivery of essentials such as food and prescriptions, while also providing emotional support to community members.^6^ Another British study demonstrated that the volunteers engagement effectively improved the population's wellbeing.^8^ Similarly, in Oman and other countries, community participation approaches were implemented during the pandemic, These approaches included empowering community members, mobilizing resources, establishing proper networking channels and disseminating information and actions at the community level.^9^

The significance of voluntary efforts becomes even more pronounced in rural or remote areas, where community support systems may be limited. Engagement in voluntary work is extensively influenced by culture. The Japanese cultural practice of ‘Osekkai’ which entails making efforts beneficial to others, played a significant role in enhancing the social capital of older people during the COVID‐19 pandemic in Japan.^10^ Voluntary service provision is a common practice in Iran, facilitated by the strong social bonds which enhance the resilience during the disasters and crises, which are prevalent in the country. For instance, in 2019, 2936 people volunteered from Yazd province for 637 crises occurred across all 31 provinces of Iran.^11^ Since the onset of COVID‐19, public volunteers have played important roles in various public health initiatives, including in public health improvement, promoting physical distancing, conducting screening and case‐finding programs, isolating affected patients, supplying necessary equipment and assisting in the recruitment of human workforce.^12^ Patient care provision in hospital settings was also among their most critical services during the COVID‐19 pandemic.^13^ A previous study in Iran revealed that volunteer activities during the pandemic typically involved educating people about COVID‐19 and related health protocols, distributing protective measures, providing economic and psychological support and assisting local authorities in implementing quarantine measures.^14^

Voluntary care provision can alleviate healthcare providers' workload, enhance care quality and partially mitigate staff shortages.^15^ It plays a pivotal role in public health promotion and constitutes a key component of clinical governance.^16^ Volunteering also improves patient satisfaction, fosters trust, reduces anxiety and negative emotions,^17^ reduces the adverse physical and mental effects of crises and facilitates disease management.^18^ Despite its positive effects, voluntary care provision is associated with challenges and concerns. For instance, volunteers often experience emotional distress due to the high mortality rate among patients during the COVID‐19 outbreak. Research has shown that coping with the death of patients is particularly challenging for volunteers participating in care programs.^18^ In another study, the lack of sufficient training regarding the characteristics of COVID‐19 and patients management was identified as a challenge that disrupts the volunteers' activities.^19^

While previous studies have extensively explored the motivations, roles, and expectations of healthcare volunteers in Iran,^12^, ^14^, ^20^, ^21^ the challenges encountered by these healthcare volunteers have not yet been thoroughly investigated. Hence, the present study aims to shed light on the challenges encountered by volunteers in the context of taking care of patients with COVID‐19, thereby making a valuable contribution to the field. Furthermore, it is noteworthy that the majority of studies worldwide exploring voluntary hospital care provision utilize quantitative designs to assess the outcomes of volunteering in various health programs.^22^, ^23^ Quantitative designs do not provide a deep understanding of volunteers' experiences. Therefore, there is a need for studies utilizing qualitative approaches to provide a better understanding of the subject. The present study was, therefore, conducted to explore the experiences of public volunteers regarding the challenges involved with voluntary hospital care provision to hospitalized patients with COVID‐19.

## 2 MATERIALS AND METHODS

### 2.1 Design

This descriptive qualitative study was carried out in 2022–2023 using inductive content analysis method.^24^, ^25^ The study's reporting adheres to the Consolidated Criteria for Reporting Qualitative Studies checklist^26^ which comprises 32 criteria developed to ensure explicit and comprehensive reporting of interviews and focus groups.^27^ Content analysis was chosen as a suitable method to organize the collected data, extract their meaning, and derive realistic conclusions.

### 2.2 Participants and setting

The study setting included 10 hospitals specialized in COVID‐19 care, located in Tehran and Shiraz, two of the largest provinces in Iran. These hospitals provided opportunities for public volunteers to attend and provide care to hospitalized patients with COVID‐19. Each hospital had between 200 and 1000 active beds, dedicated to taking care of patients with COVID‐19. These hospitals serve as the primary and largest centres for hospitalizing patients with COVID‐19, where volunteers were permitted to work. The inclusion criteria encompassed having a history of voluntary service and care provision to hospitalized patients with COVID‐19, currently volunteering at one of the study settings and expressing willingness to participate in the study and share their experiences.

A total of 18 public volunteers providing COVID‐19 care were purposefully selected in the study. The aim was to include a rich variation of participants in terms of their personal and professional characteristics such as age, gender, educational level and occupation. Upon reaching the data saturation, participant recruitment was terminated. The volunteers were informed about the possibility of volunteering through public and informal communication. Then they received an initial 2‐h training session at targeted hospitals. Education included both theoretical lessons and participatory observation of protective measures The volunteers primarily assisted the patients in meeting their general basic needs. Information about the study was provided via an in‐person meeting or a telephone conversation, followed by written information about the study and formal invitations to participate in interviews and setting interview appointments.^28^

### 2.3 Data collection

The first author collected the data from May 2022 to January 2023 through in‐depth semistructured interviews. Interviews began with general questions and progressed with specific inquiries. Examples of these questions included ‘What problems did you experience during the volunteer enrolment process, if any?’, ‘What problems did you face while providing voluntary care?’ and ‘What weaknesses did you notice in providing the voluntary care to patients?’. Moreover, probing questions such as ‘Can you elaborate on this further?’ were used to clarify ambiguities and collect more in‐depth data. At the end of the interviews, we asked participants whether they wanted to add any other point to their shared experiences. Due to ambiguities in one of the interviews, a second interview was conducted with the participant to clarify the ambiguities. Therefore, we arranged a second interview for them with their consent to obtain more data. Consequently, 21 interviews were held in total with 18 participants. The duration of the interviews ranged from 45 to 90 min. All interviews were audio recorded and transcribed verbatim. Participants were given the flexibility to choose the time and the place of the interviews.

### 2.4 Data analysis

Concurrently with data collection, we analyzed the data through Graneheim and Lundman's conventional content analysis method.^24^, ^25^, ^28^ Primarily, interviews were transcribed, and transcripts were perused several times to acquire a comprehensive understanding of them. Then, meaning units were identified and coded. The codes emerged from the data inductively and then were compared with each other and categorized based on their similarities and differences. Data collection and analysis were continued up to data saturation, that is, when no new code was obtained from the interviews. The data were managed using the MAXQDA 10 software.

### 2.5 Trustworthiness

The trustworthiness criteria of Lincoln and Guba were employed to ensure the rigour of the study.^29^ Credibility was ensured through prolonged engagement with participants and data, allocation of adequate time to data collection, and member checking. In member checking, we provided the generated codes to six participants who finally approved the congruence of our codes with their own experiences. Dependability was maintained through data collection and analysis by a team of researchers with great experience in qualitative research. To ensure confirmability, we documented all steps of the study, including data collection and data analysis, and sought input from several qualitative researchers to check the accuracy of the steps of the study. Moreover, transferability was ensured by providing clear descriptions of the study setting, sampling, participants' characteristics, data collection procedures and data analysis process.

## 3 FINDINGS

Eighteen public volunteers participated in this study, providing care to hospitalized patients with COVID‐19. The majority of participants were men (55%), with ages ranging from 23 to 55 years old. Table 1 presents the characteristics of the participants.

**Table 1:** Participants' characteristics.

No.	Gender	Age (years)	Educational level	Marital status	History of voluntary participation in previous crises
1	Male	31	Master's	Married	Yes
2	Female	28	Bachelor's	Married	No
3	Male	30	Bachelor's	Single	Yes
4	Male	29	Bachelor's	Married	No
5	Female	34	Bachelor's	Single	Yes
6	Female	30	Master's	Married	No
7	Female	23	Bachelor's	Single	Yes
8	Female	24	PhD	Married	Yes
9	Male	55	Bachelor's	Married	No
10	Female	29	Bachelor's	Single	Yes
11	Female	35	Master's	Single	No
12	Male	27	Bachelor's	Single	No
13	Male	31	Bachelor's	Married	Yes
14	Male	25	Bachelor's	Single	No
15	Female	36	Bachelor's	Single	Yes
16	Male	28	Bachelor's	Married	Yes
17	Male	34	PhD	Single	Yes
18	Male	26	Bachelor's	Single	Yes

The challenges of voluntary care provision are presented in five main categories and ten subcategories as outlined in Table 2. The categories include structural challenges, interpersonal conflicts, financial constraints, covert participation, and deteriorating condition of care provision.

**Table 2:** The main categories and subcategories of the study.

Main categories	Subcategories
Structural challenges	Lack of volunteer recruitment bases
	Ineffective organization of voluntary activities
Interpersonal conflicts	Pervasive distrust
	Heightened risk of clinical errors
Financial constraints	Conflicts between volunteer commitments and primary occupation
	Lack of financial support
Covert participation	Lack of family support
	Isolation by friends
Deteriorating condition of care provision	Mental trauma
	Physical exhaustion

### 3.1 Structural challenges

Effective voluntary care provision requires certain structural and organizational prerequisites, without which volunteers may encounter various problems and challenges. The subcategories of the structural challenges of voluntary care provision to hospitalized patients with COVID‐19 are lack of volunteer recruitment bases and ineffective organization of voluntary activities.

#### 3.1.1 Lack of volunteer recruitment bases

Participants reported that there were no certain bases for the enrollment of public volunteers and there were ambiguities in the regulations of voluntary COVID‐19 care provision and enrollment of public volunteers. They also highlighted that volunteers' efforts were not formally recognized. The participants expressed that although they wanted to volunteer since the beginning of the outbreak of the COVID‐19 disease, the hospitals refused to accept them. For the reason that before the pandemic, the necessary structures for the use of public volunteers in hospitals had not yet established.

Unfortunately, there were no structure and regulation for the enrolment of volunteers and this led to a chaos. In fact, there was no specific place for the enrolment of volunteers. (P. 17)

#### 3.1.2 Ineffective organization of voluntary activities

Hospital authorities were inadequately prepared to organize and manage volunteers as well as to supply them with necessary equipment and facilities.

We faced the shortage of equipment from the very first days of our work. For example, we had no appropriate uniforms and equipment and no place to rest. Sometimes, we had challenges with hospital authorities, particularly when there were a lot of volunteers, because the organization of the tasks and the management of the volunteers were difficult. (P. 16)

### 3.2 Interpersonal conflicts

Participants noted that healthcare providers had varying expectations and attitudes towards volunteers, influenced by their tasks, values and perspectives. The two subcategories of this category are pervasive distrust and the heightened risk of clinical errors.

#### 3.2.1 Pervasive distrust

Participants reported that healthcare providers had misperceptions about their motivations for voluntary activities and considered themselves at risk for employment loss due to volunteers' attendance at their workplace. Therefore, they had limited trust, if any, in volunteers and some of them avoided assigning care‐related tasks to them.

Staff frequently told us that we surely receive salary for care provision otherwise, we didn't accept to work among patients with COVID‐19. Some of them also clearly said that volunteers wanted to take their position and hence, did not allow them to do most of the tasks. (P. 8)

#### 3.2.2 Heightened risk of clinical errors

Participants emphasized that factors such as volunteers' limited knowledge about patient care, patient management, COVID‐19 and the basics of medicine increased the risk of clinical errors. They also noted that volunteers' limited patient care skills caused tension between volunteers and healthcare providers. Moreover, they believed that given the great importance and the high sensitivity of COVID‐19 care, volunteers' educational needs had to be assessed and fulfilled to empower them for quality COVID‐19 care provision.

It was really difficult because we entered hospital setting without any prior familiarity with patient care at hospital. Some nurses suggested that we should complete a medical emergency course before engaging in hospital care as we were not familiar with patient care and provided wrong information to patients. (P. 15)

### 3.3 Financial constraints

Volunteers faced financial challenges due to the risk of job loss and lack of a stable source of income, which hindered their ability to provide voluntary care and put them under financial strain. This category includes two subcategories: conflicts between volunteer commitments and primary occupation, and lack of financial support.

#### 3.3.1 Conflicts between volunteer commitments and primary occupation

Participants highlighted the importance of balance between voluntary activities and volunteer main occupational activities. Conflicts between voluntary and occupational activities and the managers' lack of support for the voluntary actions of the participants and emphasizing the priority of the job instead of voluntary work required some of them to select either their voluntary activities or their occupation.

The hours of voluntary hospital care conflicted with the hours of my main occupation. My boss was not flexible to allow me perform both my job duties and voluntary activities. Therefore, I was compelled to give up voluntary activities after a while. (P. 1)

#### 3.3.2 Lack of financial support

Most participants reported that they had limited access to financial support and welfare facilities. The participants acknowledged that volunteering while not providing salary, presented financial challenges and limited income, leading them two financial strain and reduced motivation for voluntary activities. Although volunteers initially started their activities without financial expectations, they later realized that continued participation came at a personal cost. For example, the volunteers had to cover commuting expenses and provide their own food supplies. Economic challenges in society and low‐income levels further diminished volunteers' motivation to continue their participation.

The hospital did not cooperate with us and did not consider a car for our commuting. I am a housewife who has no job and no income of my own. I had a lot of trouble going to the hospital. So that I had to get money from my husband regularly. Well, my husband has a low income and sometimes he reluctantly gave me money and opposed my hospital visits… (P. 13)

### 3.4 Covert participation

Participants had hidden their voluntary hospital care provision to patients with COVID‐19 from their families and friends due to the lack of their support and the high likelihood of isolation and reprimand by their families and friends. Contradictory feedback from significant others further contributed to participants' secretive participation in voluntary care provision. The two subcategories of this category are lack of family support and isolation by friends.

#### 3.4.1 Lack of family support

Most participants reported encountering disagreement from their families regarding their voluntary care provision to COVID‐19 patients. They emphasized that their families' lack of collaboration and psycho‐emotional support posed serious challenges, leading them to conceal their voluntary activities from their families. ‘My family members strongly disagreed and never allowed me to talk about this topic. Thus, I attended the hospital without informing them for the first three months’ (P. 14).

#### 3.4.2 Isolation by friends

Most participants reported the fear of isolation by their friends as a primary reason for their secret volunteering for COVID‐19 care provision at hospital. Volunteering for COVID‐19 care provision compelled participants' friends to limit their relationships with them to protect themselves against COVID‐19 affliction.

When my voluntary activities at hospital became known, I lost almost 90% of my friends, and my friends and relatives cut their relationships with me. They said that I went to a contaminated environment, thought they might be affected by COVID‐19 through me, and considered me a threat to themselves. Therefore, I preferred to hide my hospital attendance from others. (P. 12)

### 3.5 Deteriorating condition of care provision

This category encompasses the physical and mental problems experienced by participants after the care provision to hospitalized patients with COVID‐19. Examples of such problems were physical and mental fatigue, stress, and suffering due to witnessing patients' miseries. This category has two subcategories, namely mental trauma and physical exhaustion.

#### 3.5.1 Mental trauma

Participants reported experiencing a wide range of negative emotions and mental trauma due to witnessing the different negative consequences of COVID‐19, including the high death rate of the afflicted patients, their inability to manage the disease, and fear of being afflicted by it.

We were under great mental strain. Many individuals died in front of our eyes and we couldn't do anything. Moreover, I constantly experienced stress due to the potential negative consequences of my voluntary activities for myself and my family. (P.10)

#### 3.5.2 Physical exhaustion

Heavy workload, volunteer staff shortage, the necessity to wear personal protective equipment for long hours, and limited opportunity to take a rest were associated with physical exhaustion and problems for participants.

Sometimes, there was serious staff shortage, particularly during the waves of COVID‐19. The number of patients was so high that I felt I couldn't even walk due to fatigue. Wearing protective clothes and N95 mask also doubled work strain. (P.1)

## 4 DISCUSSION

The COVID‐19 disease was one of the most significant challenges disrupting the resilience of many health systems worldwide. While healthcare professionals have been the primary frontline workers combating the disease, the participation of other members of society is necessary to mitigate its consequences.^30^ Therefore, a large number of community members volunteered to offer services to hospitalized patients. The presence of volunteers in hospitals was effective in reducing the pressure on the treatment team. However, the results of some research conducted in different countries indicate that volunteers have also faced challenges in the way of voluntary participation.^31^, ^32^

The present study explored public volunteers' experiences regarding the challenges of providing voluntary hospital care to patients with COVID‐19. Findings revealed five main categories of these challenges structural challenges, interpersonal conflict, financial challenges, covert participation and deteriorating condition of care provision.

Structural challenges, namely lack of volunteer recruitment bases and ineffective organization of voluntary activities, were among the challenges of voluntary care provision to hospitalized patients with COVID‐19. In agreement with these findings, a study reported lack of an official record system for public volunteers, lack of reliable statistics about their exact number and lack of formal recognition of their activities in Iran.^33^ Another study also indicated that there was no appropriate legal framework for supporting volunteers with only 32% of national organizations being legally accountable for the outcomes of volunteers' activities.^34^ Volunteers' accomplishment depends on the formal recognition of their activities and the removal of the barriers to their practice.^35^ Therefore, healthcare authorities and policymakers need to develop policies and plans to recruit and retain volunteers and improve individuals' motivation for volunteering.^36^ In a study conducted in China, it was found that to manage, coordinate, and expand the official efforts of COVID‐19 volunteers, volunteers were identified through formal civil society organizations. Subsequently, these individuals were recruited through a mobile phone platform.^37^ Our findings also indicated ineffective management and organization of volunteers as well as a lack of necessary equipment as structural challenges of voluntary care provision to hospitalized patients with COVID‐19. Similarly, a study showed lack of effective management is a major challenge for health volunteers.^38^ Moreover, a study reported a lack of health and medical equipment as one of the most important challenges of volunteering in healthcare systems.^39^ Although voluntary activities in disaster management have great benefits, ineffective management and support reduce the effectiveness of these activities.^40^ Therefore, providing the necessary requirements and effective management are necessary to improve volunteers' motivation and satisfaction.

The second main category of the study was interpersonal conflicts with the two subcategories of pervasive distrust and the heightened risk of clinical errors. Although the participants volunteered to assist the treatment staff without any expectations, the treatment staff did not trust the volunteers and refused to accept them due to incorrect cultural perceptions. Participants encountered challenges in establishing effective communication with healthcare providers due to various factors. These included healthcare providers' lack of understanding regarding the pivotal role of volunteers in COVID‐19 management, concerns about the involvement of volunteers in specialized patient care and fear of potential job loss. Similarly, a study reported conflicts between healthcare providers and volunteers because healthcare providers perceived volunteers as a threat to their employment status.^41^ This sense of job insecurity due to voluntary activities negatively affects interpersonal relationships in healthcare settings.^42^ Healthcare providers' refusal to accept volunteers and their attempts to control them can also lead to conflicts between them.^43^ Effective interpersonal communication among healthcare providers is an essential competency for safe patient care. Therefore, healthcare providers' ineffective communication can negatively impact treatment outcomes.^44^ Furthermore, we found that volunteers limited medical knowledge and limited clinical skills contributed to interpersonal conflicts with healthcare providers which could endanger patient safety. The sudden onset of COVID‐19 necessitated healthcare authorities to recruit volunteers with inadequate clinical knowledge and skills, thereby limiting their ability to provide quality education about the basics of patient care. Therefore, volunteers faced ambiguities and conflicts in performing their roles during patient care provision. Education is a key prerequisite to the recruitment of volunteers and helps reduce the risk of clinical errors and ensure patient safety.^45^ Another study highlighted that volunteers need to have adequate skills for patient care.^46^ Therefore, formal education, supervision, and support as well as clear job descriptions are necessary for volunteers to reduce their conflicts with healthcare providers.^38^ Apparently, the improvement of volunteers' knowledge and skills can empower them and reduce interpersonal conflicts in healthcare settings.^41^

Financial constraints constituted the third main category of the challenges of voluntary care provision to hospitalized patients with COVID‐19. Study findings revealed that while volunteers helped alleviate staff shortages during the COVID‐19 pandemic, their financial challenges and problems led to a reduction in their motivation for voluntary activities. In agreement with this finding, a study showed financial problems as one of the challenges of voluntary healthcare service provision.^47^ Individuals who had fewer financial problems are more willing to participate in voluntary activities. Moreover, our findings highlighted conflicts between volunteer commitments and primary occupation. Factors such as their managers' limited collaboration with them which reduced their motivation for voluntary activities, caused fear of losing their employment and income, and required them to cease their voluntary activities. Similarly, a study reported that employees do not receive adequate organizational support for participation in voluntary activities and cannot temporarily quit their jobs.^48^ Adaptation to the requirements of a certain occupational role such as being an employee may cause difficulties in adaptation to the requirements of another role such as voluntary activities and thereby, may cause role conflict and require individuals to quit one of these roles.^49^

The third main category of the study was covert participation with the two subcategories of the lack of family support and isolation by friends. Participants concealed their voluntary care‐related activities to avoid losing family support. The fear of family members, relatives, and friends over contracting COVID‐19 led to their disapproval of volunteers' presence in hospital settings during the pandemic and necessitated isolating the volunteers. Similarly, a study reported that the lack of support from family members and significant others required some volunteers to give up their voluntary activities.^19^ Provision of education to family members and effective management of their concerns may help volunteers maintain their interpersonal relationships and improve their motivation for voluntary activities.^50^

The deteriorating condition of care provision was the last main category of the challenges of voluntary care provision to hospitalized patients with COVID‐19. Several reasons can account for volunteers' mental distress. Our findings showed that close contact with patients afflicted by COVID‐19 and its associated consequences such as bearing the emotional strain of prolonged hours looking after others' needs, witnessing patients' death and fear contraction of COVID‐19 had caused different mental challenges for volunteers. As found in research, volunteers exposed to potentially traumatic events caused by COVID‐19 experience a series of negative reactions such as anxiety, depression, and posttraumatic stress disorder.^51^ Hospital setting is a stressful environment per se and causes its attendants psychological distress^52^ and hence, volunteers for hospital care provision experience different challenges and problems such as fear and anxiety.^53^ Fear of contracting COVID‐19 and transmitting it to others reduced our participants' motivation for continuing their voluntary activities. Two previous studies also reported similar findings.^31^, ^32^ Moreover, our findings showed that besides mental problems, participants experienced physical exhaustion due to the challenging conditions of patient care, staff shortage, heavy workload and use of personal protective equipment for long hours. Consistent with this finding, a study reported that volunteers' experiences during the COVID‐19 pandemic differed from their previous experiences and were associated with physical fatigue and psychological discomfort.^14^ Furthermore, in another study, it is stated that volunteers put themselves at risk because the extreme working conditions undermine mental and physical health.^54^

## 5 LIMITATIONS

One of the study's limitations was the difficulty of identifying and recruiting eligible volunteers due to the lack of a formal system for maintaining their records. Moreover, some volunteers were reluctant to participate in the study and share their experiences. This might be understood in light of the study results which revealed some volunteers kept their volunteer work as secret and did not share it with their social network. Moreover, the qualitative design of the study limits the generalization of the findings.

## 6 CONCLUSION

The study sheds light on the challenges faced by those public volunteers in providing care to patients with COVID‐19. These challenges included a lack of a coherent system for recognizing their practice, organizational and management issues, healthcare providers' distrust, inadequate education, lack of family and social support, financial problems and limited physical and mental readiness. These challenges significantly impact volunteers' motivation for healthcare‐related voluntary activities, emphasizing the need for effective management strategies. Examples of such strategies include establishing an organizational structure within the Ministry of Health to organize and manage volunteer activities and implementing educational programs to enhance volunteers' knowledge and skills.

## AUTHOR CONTRIBUTIONS

**Fatemeh Karami**: Conceptualization; investigation; writing—original draft; methodology; writing—review and editing; formal analysis; data curation; resources; software; validation; project administration. **Alireza Nikbakht Nasrabadi**: Supervision; validation; visualization; project administration; resources; formal analysis; methodology; writing—review and editing; conceptualization. **Camellia Torabizadeh**: Methodology; validation; supervision; writing— review and editing; project administration; visualization. **Monir Mazaheri**: Methodology; validation; visualization; writing—review and editing; project administration; data curation; supervision; resources; conceptualization. **Leila Sayadi**: Funding acquisition; writing—review and editing; methodology; validation; visualization; formal analysis; project administration; data curation; supervision; resources; conceptualization; writing—original draft.

## CONFLICT OF INTEREST STATEMENT

The authors declare no conflict of interest.

## ETHICS STATEMENT

This study was conducted in accordance with the Declaration of Helsinki. The proposal for this study was approved by the Research Ethics Committees of the School of Nursing and Midwifery and Rehabilitation, Tehran University of Medical Sciences with the code: IR.TUMS.FNM.REC.1400.171. We informed participants about the study aim, voluntariness of participation in the study, freedom to withdraw at any time and confidentiality of their data. To participate in the study, verbal and informed consent was obtained from the participants.

## Data Availability

The data that support the findings of this study are available from the corresponding author upon reasonable request.
